# Unusual Presentation of a Rectovestibular Fistula as Gastrointestinal Hemorrhage in a Postmenopausal Woman

**DOI:** 10.1155/2014/578048

**Published:** 2014-12-22

**Authors:** Olga Grechukhina, Gregory M. Gressel, Graham Taylor, Jeremy I. Schwartz, Regan J. Welsh

**Affiliations:** ^1^Department of Obstetrics, Gynecology and Reproductive Science, Yale University School of Medicine, 333 Cedar Street-FMB307, New Haven, CT 06520, USA; ^2^Department of Internal Medicine, Yale University School of Medicine, 333 Cedar Street-FMB307, New Haven, CT 06520, USA

## Abstract

*Background*. Anorectal malformations (ARMs) are extremely rare and are usually identified neonatally. It is unusual for these cases to present in the postmenopausal period. This case report describes a postmenopausal patient with ARM and rectovaginal hemorrhage. *Case*. An 86-year-old, gravida 11, para 9, presented to the emergency department complaining of profuse postmenopausal vaginal bleeding. Her gynecologic history was significant only for an unclear history of an anal abnormality that was noted at birth. Speculum examination revealed profuse rectal bleeding from a rectovestibular fistula exterior to her hymenal ring. Colonoscopic examination revealed severe diverticular disease. *Conclusion*. This patient was born with an imperforate anus which resolved as rectovestibular fistula and ectopic anus. This case presents a rare clinical circumstance which integrates the fields of obstetrics, gynecology, gastroenterology, and embryology alike.

## 1. Introduction

Anorectal malformations (ARMs) are extremely rare clinical entities and are usually identified in the neonatal period. It is unusual for these cases to present in the postmenopausal period. This case report describes an atypical presentation of ARM in a postmenopausal patient with rectovaginal hemorrhage.

## 2. Case Presentation

An 86-year-old Puerto Rican woman, gravida 11, para 9, presented to the emergency room complaining of profuse vaginal bleeding. The patient reported she was in her normal state of health until a few hours prior to presentation when she had a bloody bowel movement associated with passage of bright red blood and large clots. She had been menopausal since age of 52 and she denied any history of vaginal or rectal bleeding. She had not seen a doctor in many years due to her fear of hospitals. Her past medical history was significant only for hypertension for which she was taking herbal medicine. Her obstetrical history was significant for 9 term uncomplicated vaginal deliveries in Puerto Rico. She did however report an “abnormality” with her anus which had been present since the time of her birth. Her family history was significant for an imperforate anus in her granddaughter, which was corrected at birth. She reported complete control of her own bowel movements and denied rectal incontinence but did endorse occasional mixed diarrhea and constipation. She also denied any history of urinary tract infection.

**Figure 1:**
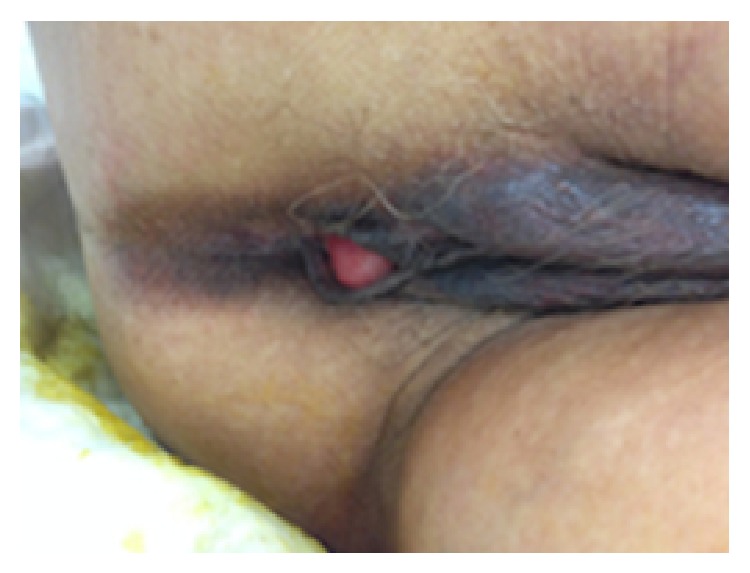
Examination demonstrating congenital absence of external anus. The rectovestibular fistula is just visible at the posterior aspect of the introitus near the fourchette.

**Figure 2:**
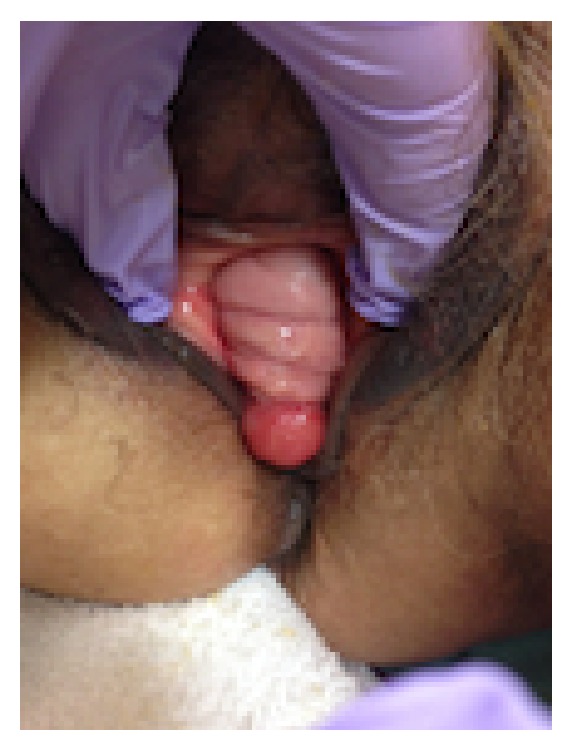
Examination demonstrating cystocele, normal vaginal tissue, and posterior rectovestibular fistula.

On admission, the patient was noted to be tachycardic (heart rate: 94–101 beats per minute) with blood pressure ranging within 164–170/84–123 mmHg. Her other vital signs were unremarkable. She was noted to be pale but had no obvious shortness of breath, chest pain, or dyspnea. A rectal examination was unable to be performed as the patient was noted to have complete absence of an external anal sphincter. The patient's laboratory values at presentation included Hb 10.7 g/dL, Hct 33.1%. The gynecologic team was called for further evaluation of possible vaginal bleeding. Bimanual examination revealed copious bright red blood from the introitus and normal postmenopausal uterus, cervix, and adnexae. However on speculum examination there was no identifiable extravasation of blood from the external cervical os. Further manual examination revealed a 2 cm opening in the posterior vaginal wall about 2-3 cm proximal to the introitus where the blood was coming from. This appeared to be a rectovaginal fistula noted caudal to the hymenal ring but within the vagina itself (Figures 1 and 2). The diagnosis of imperforate anus and subsequent ectopic anus versus rectovestibular fistula was made.

**Figure 3:**
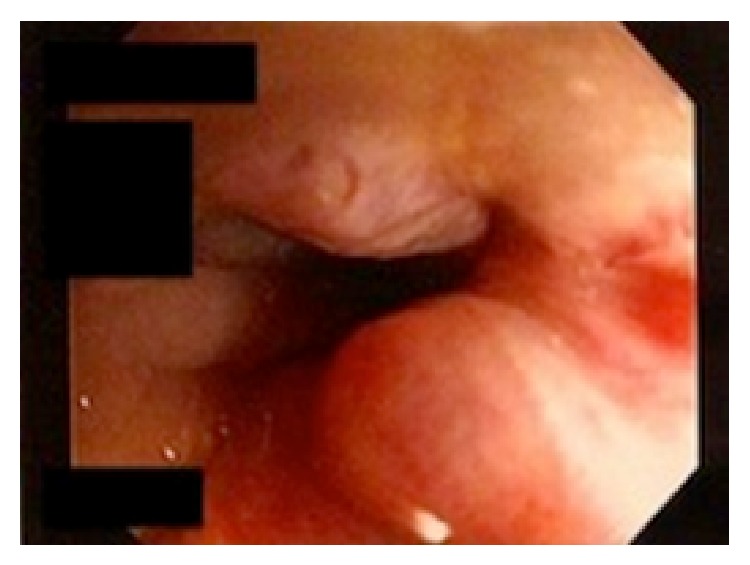
Colonoscopic image demonstrating the opening of the anorectal malformation. A small hemorrhoid is noted at the 5:00 position.

**Figure 4:**
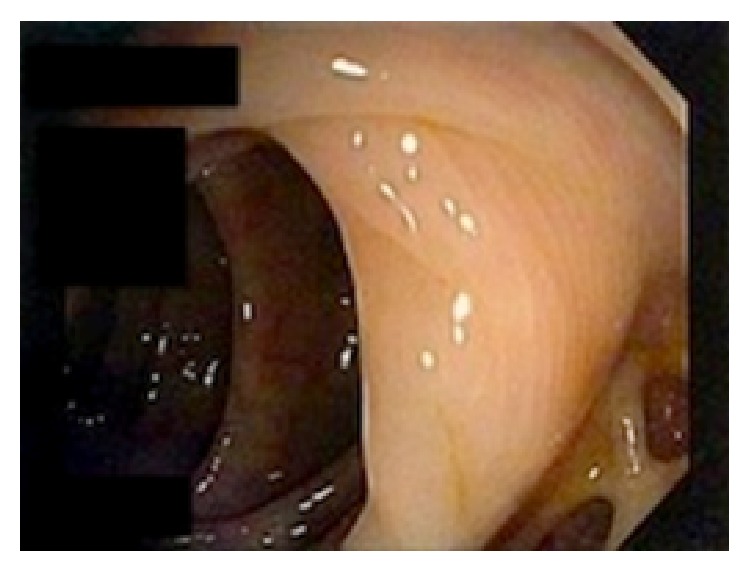
Colonoscopic image of the patient's diverticular disease. No active bleeding was noted during the colonoscopy.

Given the volume of bleeding, the patient was transferred to the intensive care unit where a tagged red blood cell study demonstrated bleeding from the sigmoid colon 8–10 cm proximal to the rectum. The patient had spontaneous cessation of her bleeding until hospital day number 5 when she had a large dark bowel movement. Interventional radiology was attempted to perform an angiography with embolization but was unable to localize the source of bleeding so an endoscopy was performed. The patient was taken for colonoscopy by the gastroenterology service where the gynecologic team assisted with introduction of a pediatric colonoscope through the fistula (Figure 3). Severe colonic diverticulosis without active bleeding was noted (Figure 4). The patient was discharged home on hospital day 9 in stable condition.

## 3. Discussion

Here we present a very rare case of an uncorrected congenital imperforate anus compensated by a rectovaginal fistula in an 86-year-old female who presented with “vaginal” bleeding. The incidence of neonatal anorectal malformations (ARMs) with imperforate anus is thought to be 1 in 4,000–5,000 live births. Rectovestibular fistula, where the opening is located distal to the hymenal ring, is the most common entity diagnosed in females, with an estimated frequency of 42% in all female newborns presenting with ARM [1]. Other less common ARMs are reported below (Table 1). More than 75% of ARMs are associated with other congenital anomalies and ~50% of cases are associated with genetic syndromes including trisomies 18 and 21, Baller-Gerold syndrome, Currarino syndrome, caudal regression syndrome, or the VACTERL association [2]. Interestingly enough, our patient had no other anomalies or evidence of genetic dysmorphology. It is believed that the vast majority of cases are identified at birth and corrected surgically [3]. The patient's own granddaughter had an imperforate anus identified at birth, which was repaired. In female patients the diagnosis of ARM after 6 months of age is considered delayed [4, 5]. Very rarely patients remain undiagnosed and untreated until adulthood [6, 7]. In these cases, patients usually present with complaints of incontinence of stool, severe constipation, or sexual dysfunction. Most of the available literature on ARM in adulthood is case reports except for one study that included 17 patients who underwent surgical correction after adolescence. The oldest patient included in this study was 55 years old. Sixty-five percent of patients were noted to have bowel control, but the majority of patients had severe constipation [6].

In the case we present, the patient was much older than the vast majority of patients with ARM. Although she was aware of an abnormal location of her anus, her symptoms (intermittent constipation and diarrhea) were not significant enough for her to present for evaluation. The fact that she had 9 spontaneous pregnancies all of which resulted in normal vaginal births is quite remarkable and indicates unaffected sexual and child-bearing functions.

Physicians caring for adult patients are not used to evaluating or diagnosing patients with congenital uncorrected ARM. This case is an example of an unusual and very late presentation of a congenital ARM with a gastrointestinal bleeding from the vagina. Gastrointestinal bleeding can result in serious morbidity and mortality and requires prompt diagnosis and management. This rare condition was not initially included in the differential diagnosis for this patient and delayed treatment of her bleeding. Management of such patients may require coordinated care from multiple teams including emergency medicine, surgical, gastroenterology, interventional radiology, intensive care, and gynecology.

## Figures and Tables

**Table 1:** Type, clinical presentation, and frequency of AVM diagnosed in female patients.

Type	Clinical presentation	Frequency
Vestibular fistula	Rectum opens immediately posterior to the vagina outside of the hymen	41%
Perineal fistula	Rectum opens immediately onto the perineum and is visible externally	28%
Imperforate anus with no fistula	No identifiable ARM noted; patient may show a “pinpoint anus”	5%
Rectal atresia and stenosis	Normal anal canal with normal sphincter mechanism	1%
Cloaca	Rectum, vagina, and urethra are fused as a single orifice	<1%

Peña and Hong, 2000 [1].
